# A scaling law of multilevel evolution: how the balance between within- and among-collective evolution is determined

**DOI:** 10.1093/genetics/iyab182

**Published:** 2021-10-23

**Authors:** Nobuto Takeuchi, Namiko Mitarai, Kunihiko Kaneko

**Affiliations:** 1 School of Biological Sciences, University of Auckland, Auckland 1142, New Zealand; 2 Research Center for Complex Systems Biology, Universal Biology Institute, University of Tokyo, Tokyo 153-8902, Japan; 3 The Niels Bohr Institute, University of Copenhagen, Copenhagen 2100-DK, Denmark; 4 Graduate School of Arts and Sciences, University of Tokyo, Tokyo 153-8902, Japan

**Keywords:** major evolutionary transitions, multilevel selection, group selection, power law, Price equation, quantitative genetics

## Abstract

Numerous living systems are hierarchically organized, whereby replicating components are grouped into reproducing collectives—*e.g.*, organelles are grouped into cells, and cells are grouped into multicellular organisms. In such systems, evolution can operate at two levels: evolution among collectives, which tends to promote selfless cooperation among components within collectives (called altruism), and evolution within collectives, which tends to promote cheating among components within collectives. The balance between within- and among-collective evolution thus exerts profound impacts on the fitness of these systems. Here, we investigate how this balance depends on the size of a collective (denoted by *N*) and the mutation rate of components (*m*) through mathematical analyses and computer simulations of multiple population genetics models. We first confirm a previous result that increasing *N* or *m* accelerates within-collective evolution relative to among-collective evolution, thus promoting the evolution of cheating. Moreover, we show that when within- and among-collective evolution exactly balance each other out, the following scaling relation generally holds: $Nm^{\alpha }$ is a constant, where scaling exponent *α* depends on multiple parameters, such as the strength of selection and whether altruism is a binary or quantitative trait. This relation indicates that although *N* and *m* have quantitatively distinct impacts on the balance between within- and among-collective evolution, their impacts become identical if *m* is scaled with a proper exponent. Our results thus provide a novel insight into conditions under which cheating or altruism evolves in hierarchically organized replicating systems.

## Introduction

A fundamental feature of living systems is hierarchical organization, in which replicating components are grouped into reproducing collectives (Maynard Smith and Szathmáry 1995). For example, replicating molecules are grouped into protocells (Joyce and Szostak 2018), organelles such as mitochondria are grouped into cells (Burt and Trivers 2006), cells are grouped into multicellular organisms (Buss 1987), and multicellular organisms are grouped into eusocial colonies (Davies *et al.* 2012; Gershwin *et al.* 2014). 

Such hierarchical organization hinges on altruism among replicating components (Bourke 2011), the selfless action that increases collective-level fitness at the cost of self-replication of individual components (West *et al.* 2007). For example, molecules in a protocell catalyze chemical reactions to facilitate the growth of the protocell at the cost of self-replication of the molecules, a cost that arises from a trade-off between serving as catalysts and serving as templates (Durand and Michod 2010; Ivica *et al.* 2013). Likewise, cells in a multicellular organism perform somatic functions beneficial to the whole organism, such as defence and locomotion, at the cost of cell proliferation due to different trade-offs (Bell 1985; Buss 1987; Kirk 1998).

Altruism, however, entails the risk of invasion by cheaters—selfish components that avoid altruism and instead replicate themselves to the detriment of a collective. For example, parasitic templates replicate to the detriment of a protocell (Maynard Smith 1979; Bansho *et al.* 2016), selfish organelles multiply to the detriment of a cell (Burt and Trivers 2006), and cancer cells proliferate to the detriment of a multicellular organism (Greaves and Maley 2012; Aktipis *et al.* 2015). Since cheaters replicate faster than altruists within a collective, they can out-compete the altruists, causing the decline of collective-level fitness—within-collective evolution, for short. However, collectives containing many altruists can reproduce faster than those containing many cheaters, so that altruists can be selected through competition among collectives—among-collective evolution. Evolution thus operates at multiple levels of the biological hierarchy in conflicting directions—conflicting multilevel evolution. Whether within- or among-collective evolution predominates exerts profound impacts on the stability and evolution of hierarchically organized replicating systems (Wilson 1975; Slatkin and Wade 1978; Aoki 1982; Crow and Aoki 1982; Leigh 1983; Kimura 1984, 1986; Frank 1994; Rispe and Moran 2000; Goodnight 2005; Traulsen and Nowak 2006; Bijma *et al.* 2007; Chuang *et al.* 2009; Leigh 2010; Frank 2012; Simon *et al.* 2013; Tarnita *et al.* 2013; Fontanari and Serva 2014; Luo 2014; Takeuchi *et al.* 2016, 2017; Blokhuis *et al.* 2018; Cooney 2019; Takeuchi and Kaneko 2019; van Vliet and Doebeli 2019), which abound in nature (Buss 1987; Maynard Smith and Szathmáry 1995; Burt and Trivers 2006; Davies *et al.* 2012; Gershwin *et al.* 2014; Joyce and Szostak 2018). Therefore, how the balance between within- and among-collective evolution is determined is an important question in biology.

Previously, we have demonstrated that the balance between within- and among-collective evolution involves a simple scaling relation between parameters of population dynamics (Takeuchi *et al.* 2016, 2017; Takeuchi and Kaneko 2019). These parameters are the mutation rate of components (denoted by *m*) and the number of replicating components per collective (denoted by *N*)—in general, *N* represents the “size” of a collective, such as the number of replicating molecules per protocell, organelles per cell, cells per multicellular organism, and organisms per colony. As *m* or *N* increases, within-collective evolution accelerates relative to among-collective evolution (*i.e.*, promoting the evolution of cheating), and *m* and *N* display the following scaling relation when within- and among-collective evolution exactly balance each other out (*i.e.*, no bias toward the evolution of cheating or altruism): $Nm^{\alpha }$ is a constant (*i.e.*, $N\propto m^{-\alpha }$), where scaling exponent *α* is approximately one half (Takeuchi *et al.* 2016, 2017; Takeuchi and Kaneko 2019). This scaling relation indicates that although *m* and *N* have quantitatively different impacts on the balance between within- and among-collective evolution, their impacts are identical if *m* is scaled with exponent *α* (*e.g.*, doubling *N* and quartering *m* approximately cancel each other out, keeping the balance of multilevel evolution).

While the above scaling relation provides a novel insight into how the balance between within- and among-collective evolution is determined, the generality of this relation is unknown because the relation has originally been demonstrated in specific models of protocells through computer simulations (Takeuchi *et al.* 2016, 2017; Takeuchi and Kaneko 2019). To shed light on the generality of the scaling relation, here, we adapt a standard model of population genetics, the Wright–Fisher model (Ewens 2004), to investigate the balance between within- and among-collective evolution. Combining computer simulations and mathematical analyses, we establish the following generalized scaling relation under the assumption that selection strengths are stationary in time: $N\propto m^{-\alpha }$, where *α* decreases to zero as selection strength *s* decreases to zero. To examine further the generality of the scaling relation, we analyze another simple model of multilevel evolution, which approaches the model studied by Kimura (1984, 1986) as s→0. Interestingly, our results show that this model displays a distinct scaling relation: $N\propto m^{-\alpha }$, where *α increases* to one as *s* decreases to zero. We show that this difference stems from the fact that our first model considers a quantitative trait, whereas our second model and Kimura’s consider a binary trait. Taken together, our results suggest that the existence of scaling relation $N\propto m^{-\alpha }$ is a general feature of conflicting multilevel evolution, but scaling exponent *α* depends on multiple factors in a nontrivial manner.

## Materials and methods

### Model

**Figure 1:**
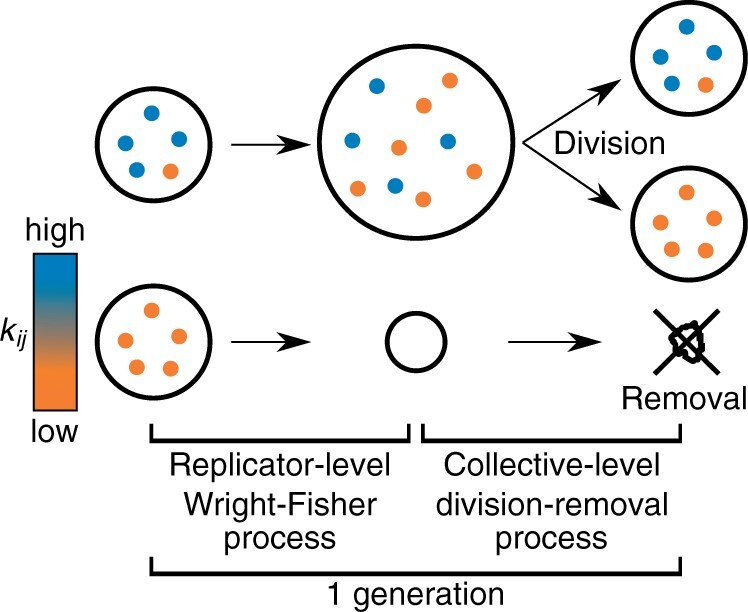
Schematic of model. Replicators (dot) are grouped into collectives (circles). *k_ij_* represents degree of altruism performed by replicators within collectives.

Our model is an extension of the Wright–Fisher model to incorporate conflicting multilevel evolution (Ewens 2004). The model consists of a population of *M* replicators grouped into collectives, each consisting of at most *N* replicators (Figure  1 and Table  1). The number of replicators in a collective can increase or decrease, and if this number exceeds *N*, the collective randomly divides into two.

**Table 1:** Symbol list

Symbol	Description
*M*	Total number of replicators.
*N*	Maximum number of replicators per collective.
*L*	Total number of collectives.
*n _i_*	Number of replicators in collective *i.*
*k_ij_*	Degree of altruism performed by replicator *j* in collective *i.*
*w_ij_*	Fitness of replicator *j* in collective *i.*
$s_{w}$	Within-collective selection strength: $-(\partial /\partial k_{ij})\ln w_{ij}$.
$s_{a}$	Among-collective selection strength: $(\partial /\partial \langle k_{i\tilde{j}}\rangle )\ln\langle w_{i\tilde{j}}\rangle$.
*m*	Probability of mutation of *k_ij_* per generation.
ϵ	Effect of mutation on *k_ij_*.
*σ*	Variance of ϵ (only in continuous-trait model).
$\langle x_{i\tilde{j}}\rangle$	Within-collective average: $n_{i}^{-1}\sum_{j=1}^{n_{i}}x_{ij}$
${\text{ave}}_{\tilde{i}}[x_{i}]$	Among-collective average: $M^{-1}\sum_{i=1}^{L}n_{i}x_{i}$
$\langle \langle x_{\tilde{i}\tilde{j}}\rangle \rangle$	Global average: $M^{-1}\sum_{i=1}^{L}\sum_{j=1}^{n_{i}}x_{ij}(={\text{ave}}_{\tilde{i}}[\langle x_{i\tilde{j}}\rangle ])$
E[X]	Expected *X* after one iteration of Wright–Fisher process.
${\text{cov}}_{i\tilde{j}}[x_{ij},y_{ij}]$	Within-collective covariance: $n_{i}^{-1}\sum_{j=1}^{n_{i}}(x_{ij}-\langle x_{i\tilde{j}}\rangle )(y_{ij}-\langle y_{i\tilde{j}}\rangle )$
${\text{cov}}_{\tilde{i}}[x_{i},y_{i}]$	Among-collective covariance: $M^{-1}\sum_{i=1}^{L}n_{i}(x_{i}-{\text{ave}}_{\tilde{i}}[x_{i}])(y_{i}-{\text{ave}}_{\tilde{i}}[y_{i}])$
$v_{w}$	Within-collective variance of *k_ij_*: ${\text{ave}}_{\tilde{i}}[\langle {\left(k_{i\tilde{j}}-\langle k_{i\tilde{j}}\rangle \right)}^{2}\rangle ]$
$v_{a}$	Among-collective variance of *k_ij_*: ${\text{ave}}_{\tilde{i}}[{\left(\langle k_{i\tilde{j}}\rangle -\langle \langle k_{\tilde{i}\tilde{j}}\rangle \rangle \right)}^{2}]$
$v_{t}$	Total variance of *k_ij_*: $\langle \langle {\left(k_{\tilde{i}\tilde{j}}-\langle \langle k_{\tilde{i}\tilde{j}}\rangle \rangle \right)}^{2}\rangle \rangle$ ($=v_{a}+v_{w}$).
$c_{w}$	Within-collective third central moment of *k_ij_*: ${\text{ave}}_{\tilde{i}}[\langle {\left(k_{i\tilde{j}}-\langle k_{i\tilde{j}}\rangle \right)}^{3}\rangle ]$
$c_{a}$	Among-collective third central moment of *k_ij_*: ${\text{ave}}_{\tilde{i}}[{\left(\langle k_{i\tilde{j}}\rangle -\langle \langle k_{\tilde{i}\tilde{j}}\rangle \rangle \right)}^{3}]$

Replicator *j* in collective *i* is assigned a heritable quantitative trait (denoted by *k_ij_*) representing the degree of altruism it performs within collective *i* (*e.g.*, *k_ij_* represents the amount of chemical catalysis a replicating molecule provides in a protocell or the amount of somatic work a cell performs in a multicellular organism). Replicators are assumed to face a trade-off between performing altruism and undergoing self-replication. Thus, the fitness of individual replicators (denoted by *w_ij_*) decreases with individual trait *k_ij_*, whereas the collective-level fitness of replicators $\langle w_{i\tilde{j}}\rangle$ increases with collective-level trait $\langle k_{i\tilde{j}}\rangle$, where $\langle x_{i\tilde{j}}\rangle$ is *x_ij_* averaged over replicators in collective *i* (*i.e.*, *x_ij_* is averaged over the index marked with a tilde; see also Table  1). For simplicity, we assume that the strengths of selection within and among collectives, defined as

(1) $$s_{w}=-\frac{\partial \ln w_{ij}}{\partial k_{ij}}\text{and}s_{a}=\frac{\partial \ln\langle w_{i\tilde{j}}\rangle }{\partial \langle k_{i\tilde{j}}\rangle },$$

respectively, depend only very weakly on *k_ij_* and $\langle k_{i\tilde{j}}\rangle$ (*i.e.*, $\partial s_{w}/\partial k_{ij}\approx 0$ and $\partial s_{a}/\partial \langle k_{i\tilde{j}}\rangle \approx 0$). This assumption implies that the relative fitness of replicators and collectives is translationally invariant with respect to *k_ij_* and $\langle k_{i\tilde{j}}\rangle$, respectively—*i.e.*, $(w_{ij}+\Delta w_{ij})/w_{ij}\approx 1+s_{w}\Delta k_{ij}$, and $(\langle w_{i\tilde{j}}\rangle +\Delta \langle w_{i\tilde{j}}\rangle )/\langle w_{i\tilde{j}}\rangle \approx 1+s_{a}\Delta \langle k_{i\tilde{j}}\rangle$. Owing to this assumption, our model informs only about short-term evolution. For computer simulations, we used the following fitness function:

(2) $$w_{ij}=e^{s_{a}\langle k_{i\tilde{j}}\rangle }\frac{e^{-s_{w}k_{ij}}}{\langle e^{-s_{w}k_{i\tilde{j}}}\rangle },$$

where $s_{w}$ and $s_{a}$ are constant so that Equation (2) satisfies the above assumption. This particular form of fitness function, however, does not affect our main conclusion, as will be seen from the fact that the mathematical analysis presented below is independent of it.

The state of the model is updated in discrete time (Figure  1). In each generation, *M* replicators are sampled with replacement from replicators of the previous generation with probabilities proportional to *w_ij_*, as in the Wright–Fisher process (Ewens 2004).

During the above sampling, a replicator inherits group identity *i* and trait *k_ij_* from its parental replicator with potential mutation (no migration among collectives is allowed). More precisely, the *k_ij_* value of a replicator is set to $k_{ij_{p}}+\epsilon$, where $k_{ij_{p}}$ is the trait of the parental replicator, and ϵ takes a value of zero with a probability of 1−m or a value sampled from a Gaussian distribution with mean zero and variance *σ* (determining mutation step size) with a probability of *m* (representing a genetic or epigenetic mutation rate). The assumption that the mean of ϵ is zero is made on the following premise: evolution is mainly driven by selection or random genetic drift, and the direction of evolution is not directly determined by mutation. Although this premise often approximates reality, it can be wrong if a mutation rate is so high as to dictate the direction of evolution as in the error catastrophe (Domingo *et al.* 2005), a situation that is ignored in this study. The assumption that *σ* is independent of *k_ij_* is made for simplicity and implies that our model informs only about short-term evolution. Although we could reduce the number of parameters by aggregating *m* and *σ* into mσ (which is the variance of ϵ), we keep them separate so that the mutation rate as usually defined is discernible.

After the above sampling, collectives containing more than *N* replicators are randomly divided into two, and those with no replicators removed (Figure  1).

### Parameter-sweep diagram

**Figure 2:**
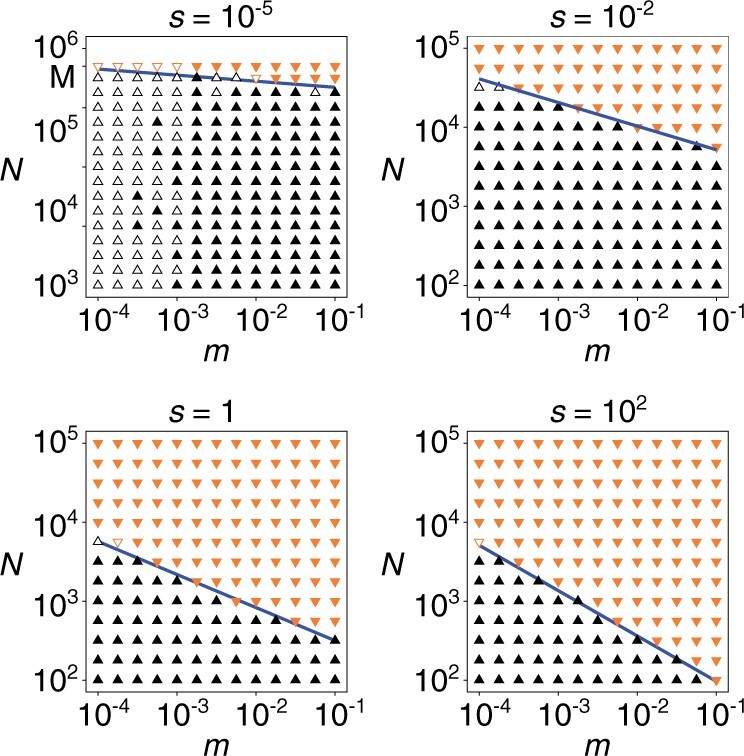
Parameter-sweep diagrams ($s_{w}=s_{a}=s,\;M=5\times 10^{5}$, and $\sigma =10^{-4}$). Symbols have following meaning: $\Delta \langle \langle k_{\tilde{i}\tilde{j}}\rangle \rangle >3\times 10^{-7}$ (black filled triangle up); $\Delta \langle \langle k_{\tilde{i}\tilde{j}}\rangle \rangle <-3\times 10^{-7}$ (orange filled triangle down); RHS of Equation (3) measured in simulations is positive (black open triangle up) or negative (orange open triangle down), where $|\Delta \langle \langle k_{\tilde{i}\tilde{j}}\rangle \rangle |<3\times 10^{-7}$. Lines are estimated boundaries where $\Delta \langle \langle k_{\tilde{i}\tilde{j}}\rangle \rangle$ changes sign (see section *Parameter-sweep diagram*).

The values of $\Delta \langle \langle k_{\tilde{i}\tilde{j}}\rangle \rangle$ used to create Figure  2 were estimated from slopes of the least squares regression of $\langle \langle k_{\tilde{i}\tilde{j}}\rangle \rangle$ against time. The estimates were unreliable if $|\Delta \langle \langle k_{\tilde{i}\tilde{j}}\rangle \rangle |<3\times 10^{-7}$ owing to a limitation of simulations, in which case the right-hand side (RHS) of Equation (3) was used as a proxy for $\Delta \langle \langle k_{\tilde{i}\tilde{j}}\rangle \rangle$.

Parameter-region boundaries across which $\Delta \langle \langle k_{\tilde{i}\tilde{j}}\rangle \rangle$ changes sign were estimated as follows. The value of *N* for which the RHS of Equation (3) becomes zero was estimated for each selected value of *m* with linear interpolation from the values of the RHS of Equation (3) (or $\Delta \langle \langle k_{\tilde{i}\tilde{j}}\rangle \rangle$ if $s_{a}\geq 10$) measured through simulations for two smallest values of *N* for which the RHS of Equation (3) (or $\Delta \langle \langle k_{\tilde{i}\tilde{j}}\rangle \rangle$ if $s_{a}\geq 10$) has different signs (the values of *m* and *V* for which simulations were run were selected as shown in Figure  2). The resulting estimates of *N* were then used to estimate the value of *α* through the least squares regression of $N\propto m^{-\alpha }$.

### Ancestor tracking

Ancestor tracking is a method that provides novel information about evolutionary dynamics by tracking the genealogy of individuals backwards in time. In our study, individuals whose genealogy was tracked were collectives. Since collectives undergo binary fission, their genealogy can be pictured as a binary tree, where an event of binary fission is represented by the coalescence of two branches of the tree. As the tree is traversed from the tips to the root (*i.e.*, from the present to the past), all branches eventually coalesce to a single branch, the stem of the tree, which represents the lineage of common ancestors of all collectives present at a particular point in time. Information about common ancestors can be visualized as time-series data along their line of descent, *i.e.*, along the stem of the tree. In Figure  5D, *n_i_* and $\langle k_{i\tilde{j}}\rangle$ of the common ancestors are plotted.

## Results

### Demonstration of a scaling relation by computer simulations

By simulating the above model, we measured the rate of change of $\langle \langle k_{\tilde{i}\tilde{j}}\rangle \rangle$, where $\langle \langle x_{\tilde{i}\tilde{j}}\rangle \rangle$ is *x_ij_* averaged over all replicators, at steady states as a function of *m* and *N*, assuming $s_{w}=s_{a}$. The result indicates the existence of two distinct parameter regions, where $\langle \langle k_{\tilde{i}\tilde{j}}\rangle \rangle$ either increases or decreases through evolution (Figure  2; the section *Parameter-sweep diagram*). (Note that although the model displays an unlimited increase or decrease of $\langle \langle k_{\tilde{i}\tilde{j}}\rangle \rangle$ over time, the model is intended to inform about short-term evolution as described above; therefore, its result should be considered as providing information about an instantaneous rate of evolution in a steady state for given parameters.)

**Figure 3:**
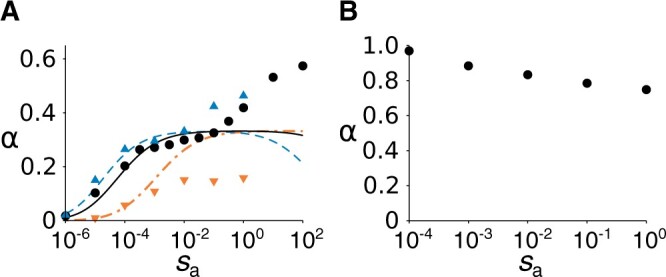
Scaling exponent *α* of parameter-region boundaries where $\Delta \langle \langle k_{\tilde{i}\tilde{j}}\rangle \rangle$ changes sign as a function of selection strength. (A) Quantitative-trait model ($M=5\times 10^{5}$ and $\sigma =10^{-4}$). Data points are simulation results (see section *Parameter-sweep diagram*). Lines are prediction by Equations (10) and (11). $s_{w}=s_{a}$ (black circle and solid line), $s_{w}=10s_{a}$ (blue triangle up and dashed line), and $10s_{w}=s_{a}$ (orange triangle down and dash-dotted line). (B) Binary-trait model ($s_{w}=s_{a}$ and $M=5\times 10^{5}$).

The two parameter regions mentioned above are demarcated by scaling relation $N\propto m^{-\alpha }$, where α↓0 as s↓0 (Figure  3A)—*i.e.*, the evolution of $\langle \langle k_{\tilde{i}\tilde{j}}\rangle \rangle$ becomes increasingly independent of *m* as *s* decreases. Similar scaling relations hold also when $s_{w}=10s_{a}$ or $s_{w}=0.1s_{a}$ (Figure  3A). These results generalize those previously obtained with specific models of protocells (Takeuchi *et al.* 2016, 2017).

### Mathematical analysis of the scaling relation

Next, we present a theory that can account for $N\propto m^{-\alpha }$ under the assumptions that $s_{a}$ and $s_{w}$ are sufficiently small. Although such a theory could in principle be built by calculating the dynamics of the frequency distribution of *k_ij_*, for simplicity, we instead calculate the dynamics of the moments of this distribution. The expected change of $\langle \langle k_{\tilde{i}\tilde{j}}\rangle \rangle$ per generation is expressed by Price’s equation as follows (Price 1972; Hamilton 1975) [see Supplementary Text S1 “Derivation of Equation (3)”]:

(3) $$\begin{array}{c} E\left[\Delta \langle \langle k_{\tilde{i}\tilde{j}}\rangle \rangle \right]={\langle \langle w_{\tilde{i}\tilde{j}}\rangle \rangle }^{-1}\{{\text{cov}}_{\tilde{i}}\left[\langle w_{i\tilde{j}}\rangle ,\langle k_{i\tilde{j}}\rangle \right]\\ +{\text{ave}}_{\tilde{i}}\left[{\text{cov}}_{i\tilde{j}}\left[w_{ij},k_{ij}\right]\right]\}, \end{array}$$

where E[x] is the expected value of *x* after one iteration of the Wright–Fisher process, ${\text{cov}}_{\tilde{i}}[x_{i},y_{i}]$ is the covariance between *x_i_* and *y_i_* over collectives, ${\text{cov}}_{i\tilde{j}}[x_{ij},y_{ij}]$ is the covariance between *x_ij_* and *y_ij_* over replicators in collective *i*, and ${\text{ave}}_{\tilde{i}}[x_{i}]$ is *x_i_* averaged over collectives (see Table  1 for precise definitions). Note that the RHS of Equation (3) is divided by $\langle \langle w_{\tilde{i}\tilde{j}}\rangle \rangle$, so that $E[\Delta \langle \langle k_{\tilde{i}\tilde{j}}\rangle \rangle ]$ depends on relative rather than absolute fitness (note also that relative fitness is independent of the absolute values of *k_ij_* and $\langle k_{i\tilde{j}}\rangle$, as described in the section *Model*).

Expanding $\langle w_{i\tilde{j}}\rangle$ and *w_ij_* in Equation (3) as a Taylor series around $\langle k_{i\tilde{j}}\rangle =\langle \langle k_{\tilde{i}\tilde{j}}\rangle \rangle$ and $k_{ij}=\langle k_{i\tilde{j}}\rangle$ (Iwasa *et al.* 1991), we obtain [see Supplementary Text S1 “Derivation of Equation (4)”]

(4) $$E\left[\Delta \langle \langle k_{\tilde{i}\tilde{j}}\rangle \rangle \right]=s_{a}v_{a}-s_{w}v_{w}+O(s_{w}^{2})+O(s_{a}^{2}),$$

where $v_{a}$ is the variance of $\langle k_{i\tilde{j}}\rangle$ among collectives, and $v_{w}$ is the average variance of *k_ij_* among replicators within a collective (Table  1). Equation (4) implies that if $s_{a}$ and $s_{w}$ are sufficiently small, the boundary of the parameter regions, on which $E[\Delta \langle \langle k_{\tilde{i}\tilde{j}}\rangle \rangle ]=0$, is given by the following equation: $s_{a}v_{a}=s_{w}v_{w}$. Since this equation is expected to imply scaling relation $N\propto m^{-\alpha }$, we need to calculate $v_{w}$ and $v_{a}$ to calculate *α*.

To calculate $v_{w}$ and $v_{a}$, we first consider a neutral case where $s_{a}=s_{w}=0$. Let the total variance be $v_{t}=v_{a}+v_{w}$. In each generation, *M* replicators are randomly sampled from replicators of the previous generation with mutation. The mutation increases $v_{t}$ to the variance of $k_{ij}+\epsilon$, which is $v_{t}+m\sigma$ since *k_ij_* and ϵ are uncorrelated (the variance of ϵ is mσ). Moreover, the sampling decreases the variance by a factor of $1-M^{-1}$ (in general, sample variance of sample size *M* is smaller than population variance by a factor of $1-M^{-1}$). Therefore, the expected total variance of the next generation is

(5) $$E\left[v_{t}^{\prime }\right]=(1-M^{-1})(v_{t}+m\sigma ).$$

Likewise, the expected within-collective variance of the next generation can be calculated as follows. To enable this calculation, we assume that all collectives always consist of $\beta^{-1}N$ replicators, where *β* is a constant (as will be described later, this approximation becomes invalid for s≳1; however, its validity for s≪1 is suggested by the fact that it enables us to calculate scaling exponent *α* correctly). Randomly sampling $\beta^{-1}N$ replicators from a collective with mutation is expected to change $v_{w}$ to

(6) $$E\left[v_{w}^{\prime }\right]=(1-\beta N^{-1})(v_{w}+m\sigma ).$$

Since $E[v_{a}^{\prime }]=E[v_{t}^{\prime }]-E[v_{w}^{\prime }]$, we obtain

(7) $$E\left[v_{a}^{\prime }\right]=(1-M^{-1})v_{a}+(\beta N^{-1}-M^{-1})(v_{w}+m\sigma ),$$

where the first term on the RHS indicates a decrease due to random genetic drift, and the second term indicates an increase due to random walks of $\langle k_{i\tilde{j}}\rangle$ through within-collective neutral evolution. Note that Equations (6) and (7) partially incorporate the collective-level division-removal process implicitly through the assumption of a constant collective size.

Next, we incorporate the effect of selection on $v_{w}$ and $v_{a}$. Allowing for the fact that replicators are sampled with probabilities proportional to fitness *w_ij_*, we can use Price’s equation to express the expected values of $v_{w}$ and $v_{a}$ after one iteration of the Wright–Fisher process as follows [see Supplementary Text S1 “Derivation of Equation (8)”; Zhang and Hill 2010]:

(8) $$\begin{array}{c} E\left[v_{w}^{\prime }\right]=\left(1-\beta N^{-1}\right)\left[v_{w}+m\sigma -s_{w}c_{w}+O(s_{w}^{2})\right]\\ E\left[v_{a}^{\prime }\right]=\left(1-M^{-1}\right)\left[v_{a}+s_{a}c_{a}+O\left(s_{a}^{2}\right)+O\left(s_{w}^{2}\right)\right]\\ \backslash \backslash +\left(\beta N^{-1}-M^{-1}\right)\left[v_{w}+m\sigma -s_{w}c_{w}+O(s_{w}^{2})\right], \end{array}$$

where $c_{w}$ is the average third central moments of *k_ij_* within a collective, and $c_{a}$ is the third central moment of $\langle k_{i\tilde{j}}\rangle$. Besides the assumption of a constant collective size, the derivation of Equation (8) involves the additional assumption that the variance of *k_ij_* within collective *i* is statistically independent of $\langle k_{i\tilde{j}}\rangle$ as *i* varies.

**Figure 4:**
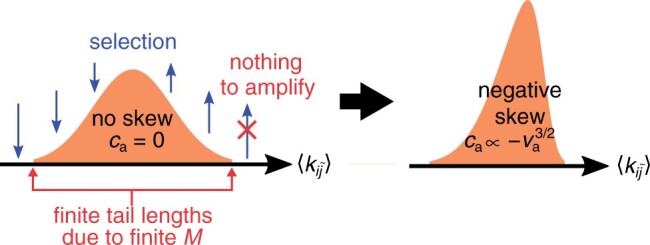
Mechanism by which trait distribution becomes skewed owing to selection and finiteness of population. Drawing depicts frequency distributions of collective-level trait $\langle k_{i\tilde{j}}\rangle$ (orange) and effect of among-collective selection (blue arrows; for simplicity, within-collective selection is not depicted). Distribution is initially assumed to be symmetric (left), so that its third central moment $c_{a}$ is zero. Tails of distribution have finite lengths due to finiteness of total population size *M* (red arrows). Because of finite lengths, left and right tails react differently to selection depending on whether they are amplified or reduced (red cross; see also main text). Consequently, distribution gets skewed (right), and $c_{a}$ becomes negative. It is postulated based on dimension that $c_{a}\propto -v_{a}^{3/2}$ at steady state, where $v_{a}$ is variance of $\langle k_{i\tilde{j}}\rangle$.

Given that the dimension of $c_{w}$ and $c_{a}$ is equivalent to that of $v_{w}^{3/2}$ and $v_{a}^{3/2}$, we make a postulate, which we verify later by simulations, that

(9) $$\begin{array}{c} c_{a}=-\gamma_{a}v_{a}^{3/2},\\ c_{w}=\gamma_{w}v_{w}^{3/2}, \end{array}$$

where $\gamma_{a}$ and $\gamma_{w}$ are positive constants. An intuitive reason for postulating that $c_{a}<0$ is due to the finiteness of *M*, as follows (Figure  4). The distribution of $\langle k_{i\tilde{j}}\rangle$ has a finite range since *M* is finite. The right tail of this distribution, the one with greater $\langle k_{i\tilde{j}}\rangle$, is exponentially amplified by selection among collectives; however, the right tail cannot be extended because its length is finite (Tsimring *et al.* 1996; Hallatschek 2011). By contrast, the left tail is contracted by among-collective selection, and this contraction is unaffected by the finiteness of the tail length. Likewise, the finiteness of tail lengths does not affect the rightward shift of the mean of the distribution due to among-collective selection. Consequently, asymmetry builds up such that the right tail becomes shorter than the left tail, hence $c_{a}<0$. The same argument can be applied to $c_{w}$, but the direction of selection is opposite, hence the opposite sign: $c_{w}>0$.

Combining Equations (8) and (9), we obtain

(10) $$E\left[v_{w}^{\prime }\right]=\left(1-\beta N^{-1}\right)\left[v_{w}+m\sigma -\gamma_{w}s_{w}v_{w}^{3/2}+O(s_{w}^{2})\right]$$

(11) $$\begin{array}{c} E\left[v_{a}^{\prime }\right]=\left(1-M^{-1}\right)\left[v_{a}-\gamma_{a}s_{a}v_{a}^{3/2}+O(s_{w}^{2})+O(s_{a}^{2})\right]\\ \backslash \backslash +\left(\beta N^{-1}-M^{-1}\right)\left[v_{w}+m\sigma -\gamma_{w}s_{w}v_{w}^{3/2}+O(s_{w}^{2})\right], \end{array}$$

Equations (10) and (11) enable us to calculate $v_{w}$ and $v_{a}$ at a steady state if $s_{a}$ and $s_{w}$ are sufficiently small (a steady state is defined as $E\left[v_{w}^{\prime }\right]=v_{w}$ and $E\left[v_{a}^{\prime }\right]=v_{a}$). For illustration, let us consider extreme conditions in which the expressions of $v_{w}$ and $v_{a}$ become simple. Specifically, if $\beta^{-1}N\gg 1$ and $s_{w}\ll {\left[\gamma_{w}\sqrt{m\sigma }{\left(\beta^{-1}N\right)}^{3/2}\right]}^{-1}$, Equation (10) implies that

(12) $$v_{w}\approx \beta^{-1}Nm\sigma .$$

Moreover, Equation (11) implies that

(13) $$M^{-1}v_{a}+\gamma_{a}s_{a}v_{a}^{3/2}\approx \left(\beta N^{-1}-M^{-1}\right)v_{w},$$

where the term involving $s_{a}M^{-1}$ is ignored under the assumption that both $s_{a}$ and $M^{-1}$ are sufficiently small (and the assumptions that $\beta^{-1}N\gg 1$ and $s_{w}\ll {\left[\gamma_{w}\sqrt{m\sigma }{\left(\beta^{-1}N\right)}^{3/2}\right]}^{-1}$ are used again). Substituting Equation (12) into Equation (13), we obtain

(14) $$v_{a}\approx \{\begin{array}{cc} Mm\sigma \left(1-\frac{\beta^{-1}N}{M}\right) & \text{if}\;s_{a}\ll {\left(\gamma_{a}\sqrt{m\sigma }M^{3/2}\right)}^{-1}\\ {\left[\frac{m\sigma }{\gamma_{a}s_{a}}\left(1-\frac{\beta^{-1}N}{M}\right)\right]}^{2/3} & \text{if}\;s_{a}\gg {\left(\gamma_{a}\sqrt{m\sigma }M^{3/2}\right)}^{-1}. \end{array}$$

Equation (14) shows that $v_{a}$ at a steady state is independent of *N* if $\beta^{-1}N\ll M$, a result that might be contrary to one’s intuition since by the law of large number, increasing *N* reduces random genetic drift within collectives and thus decelerates the growth of $v_{a}$. Indeed, the increase of $v_{a}$ per generation is approximately proportional to $N^{-1}v_{w}$ according to the second term of Equation (11). However, since $v_{w}\propto Nm$ according to Equation (12), *N* cancels out, so that $v_{a}$ is independent of *N* (see Supplementary Figure S1 for simulation results). This cancelation resembles that occurring in the rate of neutral molecular evolution, which is also independent of population size (Kimura 1968).

**Figure 5:**
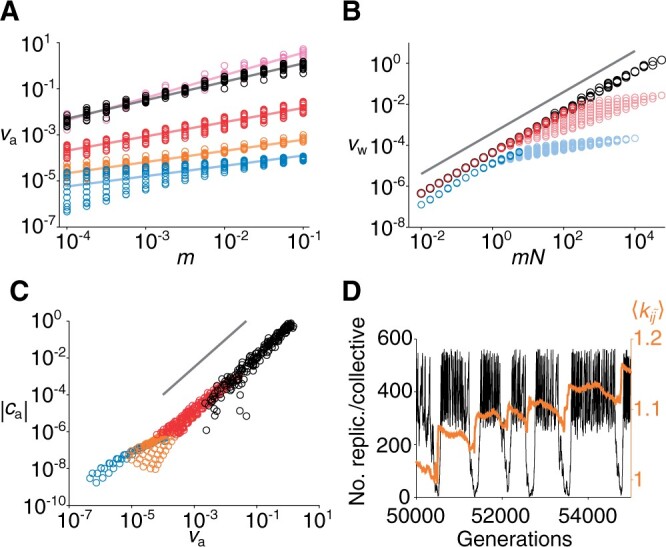
Testing theory by simulations ($M=5\times 10^{5},\;\sigma =10^{-4}$, and $s_{w}=s_{a}=s$). (A) Circles are simulation results: $s=10^{-6}$ (pink), $10^{-5}$ (black), $10^{-2}$ (red), 1 (orange), and 10^2^ (blue). Lines are least squares regression: $v_{a}\propto m^{\eta }$, where η=0.98 (pink), 0.8 (black), 0.62 (red), 0.48 (orange), and 0.45 (blue). (B) Circles are simulation results: $s=10^{-5}$ (black), $10^{-2}$ (red), and 10^2^ (blue). For $s=10^{-2}$ or 10^2^, lighter (or darker) colors indicate $\Delta \langle \langle k_{\tilde{i}\tilde{j}}\rangle \rangle <0$ (or > 0, respectively). Line is $v_{w}\propto mN$, as predicted by Equation (12). (C) Circles are simulation results: $s=10^{-5}$ (black), $10^{-2}$ (red), 10^0^ (orange), and 10^2^ (blue). Line is $|c_{a}|\propto v_{a}^{3/2}$, as postulated in Equation (9). (D) Dynamics of common ancestors of collectives: number of replicators per collective (black) and $\langle k_{i\tilde{j}}\rangle$ (orange). *s *=* *1, *N *=* *562, and *m *=* *0.01. See also section *Ancestor tracking*.

To examine the validity of Equations (10) and (11), we measured $v_{a}$, $v_{w},\;c_{a}$, and $c_{w}$ through simulations, assuming $s_{w}=s_{a}=s$ (Figure  5). The results show that $v_{a}\propto m$ for a very small value of *s* (viz., $10^{-6}$) in agreement with Equation (14) (Figure  5A). Moreover, $v_{w}\propto mN$ as predicted by Equation (12) (Figure  5B), except for cases where $\Delta \langle \langle k_{\tilde{i}\tilde{j}}\rangle \rangle <0$ (this deviation will be discussed later). Finally, $c_{a}\approx \gamma_{a}v_{a}^{3/2}$ if s≪1 (Figure  5C), and $c_{w}\approx \gamma_{w}v_{w}^{3/2}$ if s≪1 and $\Delta \langle \langle k_{\tilde{i}\tilde{j}}\rangle \rangle ≲0$ (Supplementary Figure S2), as postulated in Equation (9). Taken together, these results support the validity of Equations (10) and (11) when $s_{w}$ and $s_{a}$ are sufficiently small, and *m* and *N* are close to the boundary of the parameter regions (*i.e.*, $\Delta \langle \langle k_{\tilde{i}\tilde{j}}\rangle \rangle \approx 0$).

Using Equations (10) and (11), we can calculate the scaling exponent (*α*) of the boundary of the parameter regions for sufficiently small $s_{a}$ and $s_{w}$. Since $E[\Delta \langle \langle k_{\tilde{i}\tilde{j}}\rangle \rangle ]=0$ on the parameter boundary, Equation (4) implies that $v_{a}/v_{w}\approx s_{w}/s_{a}$. Thus, for extreme parameter conditions (viz., $1\ll \beta^{-1}N\ll M$, and $s_{w}\ll {\left[\gamma_{w}\sqrt{m\sigma }{\left(\beta^{-1}N\right)}^{3/2}\right]}^{-1}$), Equations (12) and (14) imply that

(15) $$\begin{array}{cc} \alpha \approx 0 & \text{if}\;\;s_{a}\ll {\left(\gamma_{a}\sqrt{m\sigma }M^{3/2}\right)}^{-1}\\ \alpha \approx 1/3 & \text{if}\;\;s_{a}\gg {\left(\gamma_{a}\sqrt{m\sigma }M^{3/2}\right)}^{-1}. \end{array}$$

For $s_{a}∼{\left(\gamma_{a}\sqrt{m\sigma }M^{3/2}\right)}^{-1}$, Equations (12) and (13) imply that

(16) $$rM^{-1}\beta^{-1}\left(N+\sqrt{r}s_{a}\Gamma N^{3/2}m^{1/2}\right)\approx 1,$$

where $r=s_{w}/s_{a}$ and $\Gamma =\gamma_{a}\sqrt{\sigma /\beta }M$, and Equation (16) implies that *α* increases from zero to one-third as $\sqrt{r}s_{a}$ increases from zero.

We also numerically obtained *α* by calculating the values of *N* and *m* ($m\in [10^{-4},10^{-1}]$) that satisfy $v_{a}/v_{w}=s_{w}/s_{a}$ at a steady state using Equations (10) and (11), and the values of *β*, $\gamma_{a}$, and $\gamma_{w}$ estimated from Figure 5BC and Supplementary Figure S2, respectively [viz., $\beta^{-1}=0.45$ and $\gamma_{a}=0.26$ through least squares regression of Equations (12) and (9) for $s=10^{-6}$ and $10^{-2}$, respectively; $\gamma_{w}=0.25$ through least squares regression of Equation (9) for $\Delta \langle \langle k_{\tilde{i}\tilde{j}}\rangle \rangle ≲0$]. The results agree with the simulation results for $s_{a}<1$ when *r *=* *1 and 10, and for $s_{a}<10^{-3}$ when *r *=* *0.1 (Figure  3A). We do not know why the validity of analytical prediction is restricted when *r* is small. Overall, the above results support the validity of Equations (10) and (11) for sufficiently small values of $s_{a}$ and $s_{w}$.

In addition, we note that the postulate in Equation (9) is also supported by previous studies calculating the evolution of a quantitative trait (viz., fitness) subject to single-level selection (Tsimring *et al.* 1996; Hallatschek 2011). These studies show that fitness increases through evolution at a rate proportional to the two-third power of the mutation rate. That result is consistent with Equations (10) and (11) and, hence, also with Equation (9), as follows. Since Tsimring *et al.* (1996) assume single-level selection and a very large population, let us also assume that $s_{w}=0$ and M→∞, respectively, in our model. Then, Equations (4) and (14) imply that logarithmic fitness, $\ln\langle \langle w_{\tilde{i}\tilde{j}}\rangle \rangle \propto \langle \langle k_{\tilde{i}\tilde{j}}\rangle \rangle$, increases at a rate proportional to $m^{2/3}$ (Supplementary Figure S1). Reversing the argument, we can also use the model of Tsimring *et al.* (1996) to estimate the value of $\gamma_{a}$ as about 0.25 (Supplementary Text S1 under “Estimation of $\gamma_{a}$”), which matches the value measured in our model (viz., 0.26). Moreover, the model of Tsimring *et al.* (1996) can also be applied to estimate $\gamma_{w}$, and the value of $\gamma_{w}$ measured in our model is about 0.25 (Supplementary Figure S2). Taken together, these agreements corroborate the validity of Equation (9).

Finally, to clarify why Equations (10) and (11) deviate from the simulation results for s≳1 or $\Delta \langle \langle k_{\tilde{i}\tilde{j}}\rangle \rangle <0$, we tracked the genealogy of collectives backwards in time to observe the common ancestors of all collectives (the section *Ancestor tracking*). Figure  5D displays the dynamics of $\langle k_{i\tilde{j}}\rangle$ and *n_i_* (the per-collective number of replicators) in these ancestors along their single line of descent. The results indicate that the model displays a phenomenon previously described as *evolutionarily stable disequilibrium* (ESD, for short; Takeuchi *et al.* 2016). Briefly, the collectives constantly oscillate between growing and shrinking phases (Figure  5D). During the growing phase, the collectives continually grow and divide, and their $\langle k_{i\tilde{j}}\rangle$ values gradually decline through within-collective evolution, a decline that eventually puts the collectives to a shrinking phase. In the shrinking phase, the collectives steadily decrease in the number of constituent replicators; however, their $\langle k_{i\tilde{j}}\rangle$ values abruptly jump at the end of the shrinking phase, a transition that brings the collectives back to the growing phase. This sudden increase of $\langle k_{i\tilde{j}}\rangle$ is due to random genetic drift induced by very severe within-collective population bottlenecks. Although such an increase of $\langle k_{i\tilde{j}}\rangle$ is an extremely rare event, it is always observed in the lineage of common ancestors because these ancestors are the survivors of among-collective selection, which favors high $\langle k_{i\tilde{j}}\rangle$ values (Takeuchi *et al.* 2016, 2017).

ESD breaks the assumption—involved in Equations (10) and (11)—that all collectives always consist of $\beta^{-1}N$ replicators because ESD allows extremely small collectives to regrow and contribute significantly to $v_{w}$ and $v_{a}$ (note that the contributions of collectives to $v_{w}$ and $v_{a}$ are proportional to the number of replicators they contain, as defined in Table  1). We found that ESD occurs for s≳1 (Figure  5D) or for $\Delta \langle \langle k_{\tilde{i}\tilde{j}}\rangle \rangle <0$ (Supplementary Figure S3). Thus, ESD might be responsible for the failure of Equations (10) and (11) to predict *α* for s≳1 (Figure  3) as well as the fact that $c_{a}\neq \gamma_{a}v_{a}^{2/3}$ for s≳1 (Figure  5C). In addition, ESD might also be responsible for the fact that $v_{w}$ is not proportional to *mN* when $\Delta \langle \langle k_{\tilde{i}\tilde{j}}\rangle \rangle <0$ (Figure  5B).

**Figure 6:**
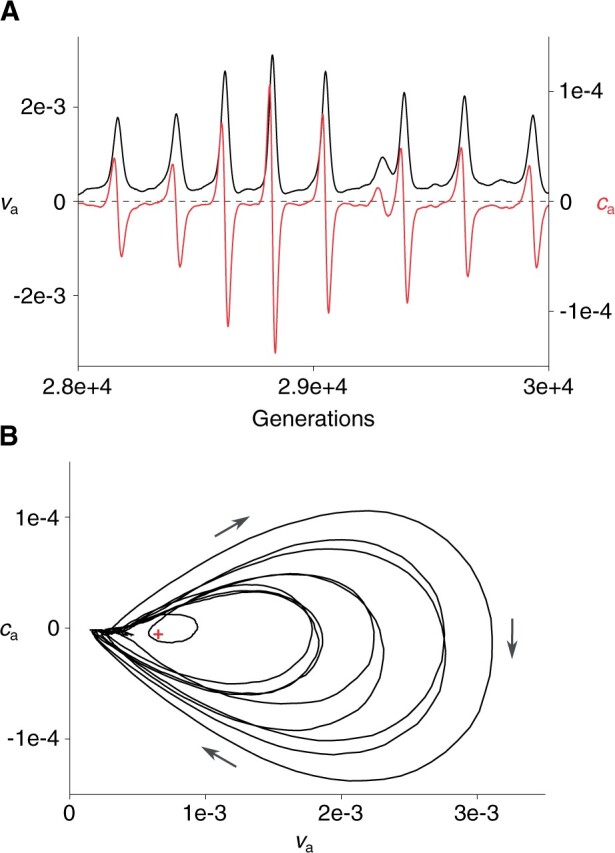
Oscillation of $v_{a}$ and $c_{a}$ observed in simulations (*N *=* *5623, *m *=* *0.1, $s_{a}=s_{w}=1,\;M=5\times 10^{5}$, and $\sigma =10^{-4}$). (A) $v_{a}$ (black, left coordinate) and $c_{a}$ (red, right coordinate) as functions of generations. (B) Phase-space trajectory of same data as shown in A. Cross indicates mean values of $v_{a}$ and $c_{a}$ in this trajectory. Arrows indicate direction of trajectory.

Another potential reason for the failure of Equations (10) and (11) for s≥1 is the fact that $v_{a}$ and $c_{a}$ constantly oscillate with a periodic sign change of $c_{a}$ (Figure  6). This oscillation not only invalidates the assumption that $c_{a}=\gamma_{a}v_{a}^{2/3}$ but also makes it questionable to consider a steady-state solution of Equations (10) and (11). Finally, we add that this oscillation is distinct from ESD, in that it is observed in terms of $v_{a}$ and $c_{a}$, which are properties of an entire population of collectives, whereas ESD is observed in terms of the properties of common ancestors of collectives.

### Comparison to a binary-trait model

To examine further the generality of the scaling relation described above, we next consider a study by Kimura (1984, 1986). Kimura has investigated a binary-trait (*i.e.*, two allele) model of multilevel evolution formulated based on a diffusion equation. Using this model, Kimura has revealed the following scaling relation that holds when within- and among-collective evolution exactly balances each other out:

(17) $$N=\frac{\beta s_{a}}{4s_{w}}m^{-1}$$

(the notation has been converted to ours as described in Supplementary Text S1 under “Converting Kimura’s notation into ours”; Kimura 1984, 1986). Equation (17) is derived under the assumption that the steady-state frequency of the altruistic allele is identical to that in the absence of selection, thus involving a weak-selection approximation (Kimura 1984, 1986). Therefore, the scaling exponent in Kimura’s model (α≈1) differs from that in ours (α≈0) for $s_{a}\approx 0$ and $s_{w}\approx 0$.

To study how *α* depends on *s* (where $s=s_{w}=s_{a}$) if the trait is binary, we modified our model into a binary-trait model by assuming that *k_ij_* switches between zero and one at mutation rate *m*. By simulating the modified model, we obtained a parameter-sweep diagram, where parameter regions were defined by the sign of $\langle \langle k_{\tilde{i}\tilde{j}}\rangle \rangle -1/2$ at steady states (Supplementary Figure S4; this definition of parameter regions is essentially equivalent to that used for the quantitative-trait model, in that it can be rephrased in terms of the sign of $\Delta \langle \langle k_{\tilde{i}\tilde{j}}\rangle \rangle$ at $\langle \langle k_{\tilde{i}\tilde{j}}\rangle \rangle =1/2$). The results show that the parameter-region boundary constitutes scaling relation $N\propto m^{-\alpha }$, where α↑1 as s↓0 (Figure  3B)—*i.e.*, the evolution of $\langle \langle k_{\tilde{i}\tilde{j}}\rangle \rangle$ becomes increasingly dependent on *m* as *s* decreases. Therefore, the way *α* depends on *s* is compatible with Equation (17), but is opposite to that in the quantitative-trait model, where α↓0 as s↓0 (Figure  3A).

To pinpoint why the two models yield such distinct predictions, we re-derived Equation (17) using the method developed in the section *Mathematical analysis of the scaling relation* (for details, see Supplementary Text S1 under “Derivation of Kimura’s result through our method”). Briefly, the most important difference from the quantitative-trait model is in the definition of mutation: ϵ depends on *k_ij_* in the binary-trait model (specifically, ϵ takes a value of $1-2k_{ij_{p}}$ with a probability of *m*, where $k_{ij_{p}}$ is the trait of a parental replicator). While this difference does not alter the condition for a parameter-region boundary implied by Equation (3), it significantly changes the calculation of variances. Namely, Equations (6) and (7) need to be modified to

(18) $$E\left[v\prime_{w}\right]\approx (1-\beta N^{-1})\left[v_{w}+4m(1-m)v_{a}\right]$$

(19) $$E\left[v\prime_{a}\right]\approx (1-M^{-1})v_{a}+(\beta N^{-1}-M^{-1})v_{w}$$

(20) $$\backslash \backslash \backslash \backslash \backslash -4(1-\beta N^{-1})m(1-m)v_{a},$$

respectively, where we have assumed that the parameters are on a parameter-region boundary and, therefore, that $\langle \langle k_{\tilde{i}\tilde{j}}\rangle \rangle =1/2$. Equations (18) and (19) ignore the effect of selection and are thus an approximation expected to be valid for sufficiently weak selection. Dividing Equation (18) by Equation (19) on each side and assuming a steady state (*i.e.*, $E\left[v\prime_{w}\right]/E\left[v\prime_{a}\right]=v_{w}/v_{a}$), we obtain

(21) $$\frac{v_{w}}{v_{a}}\approx \frac{4m(1-m)(1-\beta N^{-1})}{\beta N^{-1}-M^{-1}}.$$

Imposing the condition for a parameter-region boundary, $v_{w}/v_{a}\approx s_{a}/s_{w}$, we obtain

(22) $$\frac{4m(1-m)(1-\beta N^{-1})}{\beta N^{-1}-M^{-1}}\approx \frac{s_{a}}{s_{w}},$$

which is approximately the same as Equation (17) if m≪1 and $M^{-1}\ll \beta N^{-1}\ll 1$ as assumed by Kimura (1984, 1986).

Equations (18) and (19) allow us to understand why the two models display different scaling exponents. These equations contain terms involving $\pm 4m(1-m)v_{a}$, which increase $v_{w}$ and commensurately decrease $v_{a}$. This “transfer” of variance occurs because mutation causes $\langle k_{i\tilde{j}}\rangle$ to tend toward 1/2, for which $v_{w}$ is maximized, in every collective. In other words, mutation directly causes the convergent evolution of $\langle k_{i\tilde{j}}\rangle$, raising the $v_{w}/v_{a}$ ratio. Consequently, the balance between within- and among-collective evolution strongly depends on *m*. By contrast, the quantitative-trait model assumes that mutation does not cause any directional evolutionary change in $\langle k_{i\tilde{j}}\rangle$. Moreover, mutation equally increases $v_{w}$ and $v_{a}$ according to Equations (10–12). Consequently, the balance between within- and among-collective evolution does not much depend on *m* if selection is weak.

## Discussion

The results presented above suggest that scaling relation $N\propto m^{-\alpha }$ is a general feature of conflicting multilevel evolution. Scaling exponent *α*, however, depends in a nontrivial manner on the strength of selection and whether altruism is a quantitative or binary trait.

Although we have assumed that the parameters involved in the scaling relation—the mutation rate, selection strength, and the distinction between quantitative and binary traits—are independent of each other, these parameters are potentially correlated in reality. While such correlations are not well understood (Thompson *et al.* 2013; Kasper *et al.* 2017), discussing them can illustrate the utility of the findings of this study. For this illustration, we first note that whether altruism is a quantitative or binary trait can be translated into the number of loci involved in altruism: a quantitative trait involves many loci, whereas a binary trait involves one. The number of loci is likely to be positively correlated with the mutation rate of the trait, and it is possibly negatively correlated with the effect size of mutation (*e.g.*, a single locus with large effects *vs* many loci with small effects). The effect size of mutation, in turn, is possibly positively correlated with the strength of selection. These correlations, which we assume here for the sake of illustration, would imply a spectrum of altruism ranging from a strongly selected, binary trait with a low mutation rate to a weakly selected, quantitative trait with a high mutation rate (we are ignoring the possibility that mutations have highly heterogeneous effects). Such correlations would be conducive to the evolution of altruism, an inference that is enabled by the following findings of this study: binary-trait altruism is susceptible to the invasion by cheaters for a high mutation rate, but this susceptibility decreases with selection strength (*α* decreases with *s*); by contrast, quantitative-trait altruism is relatively insensitive to mutation for weak selection (*α* decreases to zero as *s* decreases to zero).

Although the results of this study are phrased in the language of multilevel selection (Wilson 1975; Slatkin and Wade 1978; Aoki 1982; Crow and Aoki 1982; Leigh 1983; Kimura 1984, 1986; Frank 1994; Rispe and Moran 2000; Goodnight 2005; Traulsen and Nowak 2006; Bijma *et al.* 2007; Chuang *et al.* 2009; Leigh 2010; Frank 2012; Simon *et al.* 2013; Tarnita *et al.* 2013; Fontanari and Serva 2014; Luo 2014; Takeuchi *et al.* 2016, 2017; Blokhuis *et al.* 2018; Cooney 2019; Takeuchi and Kaneko 2019; van Vliet and Doebeli 2019), they can easily be rephrased, mutatis mutandis, in the language of kin selection (Cheverud 1985; Queller 1985; Queller 1992; Wolf *et al.* 1999; Frank 1997; Gardner *et al.* 2007; Bijma and Wade 2008; Chuang *et al.* 2010; McGlothlin *et al.* 2010; Queller 2011; Akçay and van Cleve 2012; McGlothlin *et al.* 2014). To do this, we define the relatedness of replicators as the regression coefficient of $\langle k_{i\tilde{j}}\rangle$ on *k_ij_* (Hamilton 1970; Rice 2004), *i.e.*, $R=v_{a}/(v_{a}+v_{w})$, and express all the results in terms of *R* instead of $v_{w}/v_{a}$. Therefore, our results are compatible with the kin selection theory.

An important issue to address for future research is to test whether the scaling relation is observed in reality. Such tests could in principle be conducted through evolutionary experiments.

Capturing the essence of multilevel selection, the models and analyses presented above are likely to have broad utility. They are generally relevant for the evolution of altruism in replicators grouped into reproducing collectives, *e.g.*, symbionts, organelles, or genetic elements grouped into cells (Burt and Trivers 2006), cells grouped into multicellular organisms (Buss 1987), or other systems that have emerged through major evolutionary transitions (Maynard Smith and Szathmáry 1995).

## Data availability

Supplementary Texts and Figures can be found in Supplementary File S1. C++ source code implementing the models can be found in Supplementary File S2. Supplementary material is available at figshare: https://doi.org/10.25386/genetics.14337251.
